# Reconstruction of complex shallow defects of the extremities with free serratus musculofascial flap*

**DOI:** 10.3906/sag-1907-57

**Published:** 2020-02-13

**Authors:** Hakan ÇİNAL, Ensar Zafer BARIN, Murat KARA, Kerem YILMAZ, Harun KARADUMAN, İhtişam Zafer CENGİZ, Önder TAN

**Affiliations:** 1 Department of Plastic, Reconstructive, and Aesthetic Surgery, Faculty of Medicine, Atatürk University, Erzurum Turkey; 2 Department of Plastic, Reconstructive, and Aesthetic Surgery, Gaziosmanpaşa Medicalpark Hospital İstanbul / Turkey

**Keywords:** Defect, extremity, fascia, free flap, serratus

## Abstract

**Background/aim:**

Thin and flexible flaps are needed for the repair of extremity tissue defects. Serratus fascia flaps are the most suitable options. There are only a few case reports and case series in the literature. We have aimed to increase the success rate by taking the serratus fascia flaps together with some muscle tissue for the repair of extremity tissue defects.

**Materials and methods:**

Between 2006 and 2015, 12 free serratus musculofascial flaps (FSMFFs) were transferred to 11 patients (8 males, 3 females) who had tissue shallow defects of the extremities due to different etiologic factors. The mean age was 24.6 years. Hospital records and patient photographs were reviewed and age, sex, etiologic cause, follow-up period, complication, flap success, cosmetic appearance, and functional results of the extremity were examined.

**Results:**

The mean follow-up period was 29.5 months (7–109 months). All of the flaps survived fully and no partial or full necrosis was observed. Partial graft loss was encountered in one patient and it was recovered secondarily with wound dressing. A major deficiency was not experienced postoperatively in the functions and movements of the extremities retained. The cosmetic appearance of the operation area was acceptable or fine. The donor areas healed in all the patients without any problems, and the scars were hidden and inconspicuous.

**Conclusion:**

In reconstruction of complex shallow defects of the extremities in which a gliding effect is desired, FSMFF may be an ideal option with its advantages as it does not sacrifice a major vessel, does not leave a hidden and short scar, can be harvested in wide dimensions, and allows the use of the surrounding tissues such as the latissimus dorsi muscle and scapular bone.

## 1. Introduction

Reconstruction of complex superficial defects of the extremities like tendon, bone, and vessel, which leave the vital structures open, is difficult as it requires a flexible approach enabling a gliding effect, as well as thin tissue, thus not defiling the cosmetic appearance and movements due to its size. Reconstructive options are limited since the donor areas from which thin flaps can be taken are not many, as well as the fact that larger flaps cannot be obtained, and furthermore there is morbidity of the donor area. For this purpose, radial forearm flaps [1], dorsalis pedis flaps [2], and thinned anterolateral thigh flaps [3] as skin flaps and temporoparietal [4], radial forearm [5], lateral arm [6], and scapular flaps [7] as fascial flaps have been used. Thin flaps are needed for the best results in the mobile parts of the body. Although a free serratus fascia flap (FSFF) is a very good option in this regard, there are few studies in the literature regarding this option [8–12]. Flaps can be taken together with the muscle to increase the chances of success. This increases the success but makes the flap thicker and noncosmetic. For better cosmetic and functional results, taking the flap only with the fascia is another option. However, the possibility of damaging the subfascial plexus will increase when the flap is raised. In this study, we obtained the advantages of both approaches and took the flap with very thin muscle tissue to avoid its disadvantages. Thus, we aimed to increase the flap’s success without causing any important cosmetic or functional problems.

However, no ideal thin flap is available that can be used for reconstruction of shallow extremity defects. In this study, as one of the largest series in extremities, a free serratus musculofascial flap (FSMFF) has been used in the coverage of lower and upper extremity superficial defects, and its outcomes are discussed.

## 2. Materials and methods

The study was approved by the Atatürk University Medical Faculty’s Ethical Council for Clinical Research with ethical council number 2019/04–42. Between 2006 and 2015, 12 FSMFFs were transferred to 11 patients (8 males, 3 females) who had tissue shallow defects of the extremities due to different etiologic factors. Postoperatively, observations were performed every hour for the first four days. Observations included hand Doppler signal, color, and temperature. Additionally, bleeding of the flap was also performed in critical cases. Hospital records and patient photographs were reviewed and age, sex, etiologic cause, follow-up period, complications, flap success, cosmetic appearance, and functional results of the extremity were examined.

### 2.1. Surgical technique

Under general anesthesia, the defect was revealed in the recipient area following surgical debridement. Once the recipient vessels were prepared for microanastomosis, FSMFF dissection was started. The arm was taken to 90° abduction in the supine or lateral decubitus position, depending on the defect location. The axillary apex and posterior axillary line were marked. On the lateral thorax wall between the 5th and 9th Costas in front of the posterior axillary line, flap boundaries in the planned dimensions were determined. As a rule, flaps were planned as to be at least 2 cm larger than the defect sizes. The anterior side of the latissimus dorsi muscle was found after reaching the skin and subcutaneous tissues through a vertical incision of 10 cm in adults and 5–6 cm in children, on the posterior axillary line. The thoracodorsal neurovascular pedicle was found beneath the muscle by elevating it backwards. The serratus branch was reached and suspended by dissecting the neurovascular pedicle 5 to 6 cm proximally. The serratus fascia was then elevated based on its vascular pedicle as an island flap in the planned dimension with blunt scissors by including the underlying serratus muscle in a split manner under frequent bleeding control. During dissection, the long thoracic nerve, which is the motor nerve of the serratus muscle, was not seen. The flap was allowed to perfuse for about 15 min. The flap was then harvested and transferred to the recipient site, after dividing the serratus branch from the main thoracodorsal vessel. The microvascular anastomosis was performed under a microscope following the inset of the flap. The end-to-side technique and 10-0 microsutures were used to avoid size discrepancy, because the diameters of donor vessels were 1 to 1.5 mm in average. Depending on the defect location, the radial or ulnar vessels in the upper extremity and the posterior tibial or dorsalis pedis vessels in the lower extremity were used as the recipient vessels. After perfusion was obtained, a Penrose drain was placed under the flap, which was covered with a split thickness skin graft following bleeding control and placement of a Jackson–Pratt drain. Subsequently, the donor site was covered primarily and then the operation was ended.

## 3. Results

The mean follow-up period was 29.5 months (7–109 months). Patients were between the ages of 3 and 46 years old (mean: 24.6 years old). The etiologies were crush injuries due to traffic accidents in 5 patients, electrical burns in 3 patients, contact burns in 2 patients, and skin necrosis following local anesthetic infiltration in 1 patient. Nine of the 12 defects were located on the feet and 3 were on the hands. Flap dimensions were 7 × 6 and 16 × 11 cm, while the vascular pedicle lengths varied between 3 and 8 cm (Table).

All the flaps survived, and no partial or total necrosis was observed. A partial graft loss was seen in one patient and it was recovered subsequently with medical dressing. No patients had bleeding, hematoma, arterial or venous microthrombosis, wound dehiscence, or infection; hence, early or late revision was not required. There were reasonable functions and motions of the affected extremities after surgery. We did not receive any negative responses from patients about the cosmetic appearance of the flaps. The donor sites healed uneventfully with inconspicuous scars in all patients. We did not observe any abnormal skin changes on the flap in the follow-up period. None of the patients developed “wing scapula” deformity due to long thoracic nerve damage.

**Table 1:** Summary of the case series.

Patientno.	Age	Sex	Etiology	Defectlocation	Flap size(cm)	Pediclelength (cm)	Complications	Follow-up (months)
1	11	F	Traffic accident	Left foot	10 × 8	3	-	44
2	14	M	Traffic accident	Left foot	15 × 10	8	-	72
3	20	M	Electrical burn	Right foot	11 × 5	8	-	9
4	27	M	Local anesthetics	Right foot	12 × 5	5	-	7
4	27	M	Local anesthetics	Left foot	12 × 5	5	-	7
5	28	M	Traffic accident	Left foot	12 × 10	8	-	15
6	30	M	Electrical burn	Left foot	10 × 9	6	-	10
7	30	F	Traffic accident	Right hand	10 × 7	5	-	109
8	34	M	Electrical burn	Right hand	7 × 6	5	-	9
9	25	M	Contact burn	Right foot	15 × 8	6	-	26
10	3	M	Traffic accident	Right foot	11 × 6	5	Partial graft loss	8
11	46	F	Contact burn	Left hand	16 × 11	5	-	16

**Figure 1:**
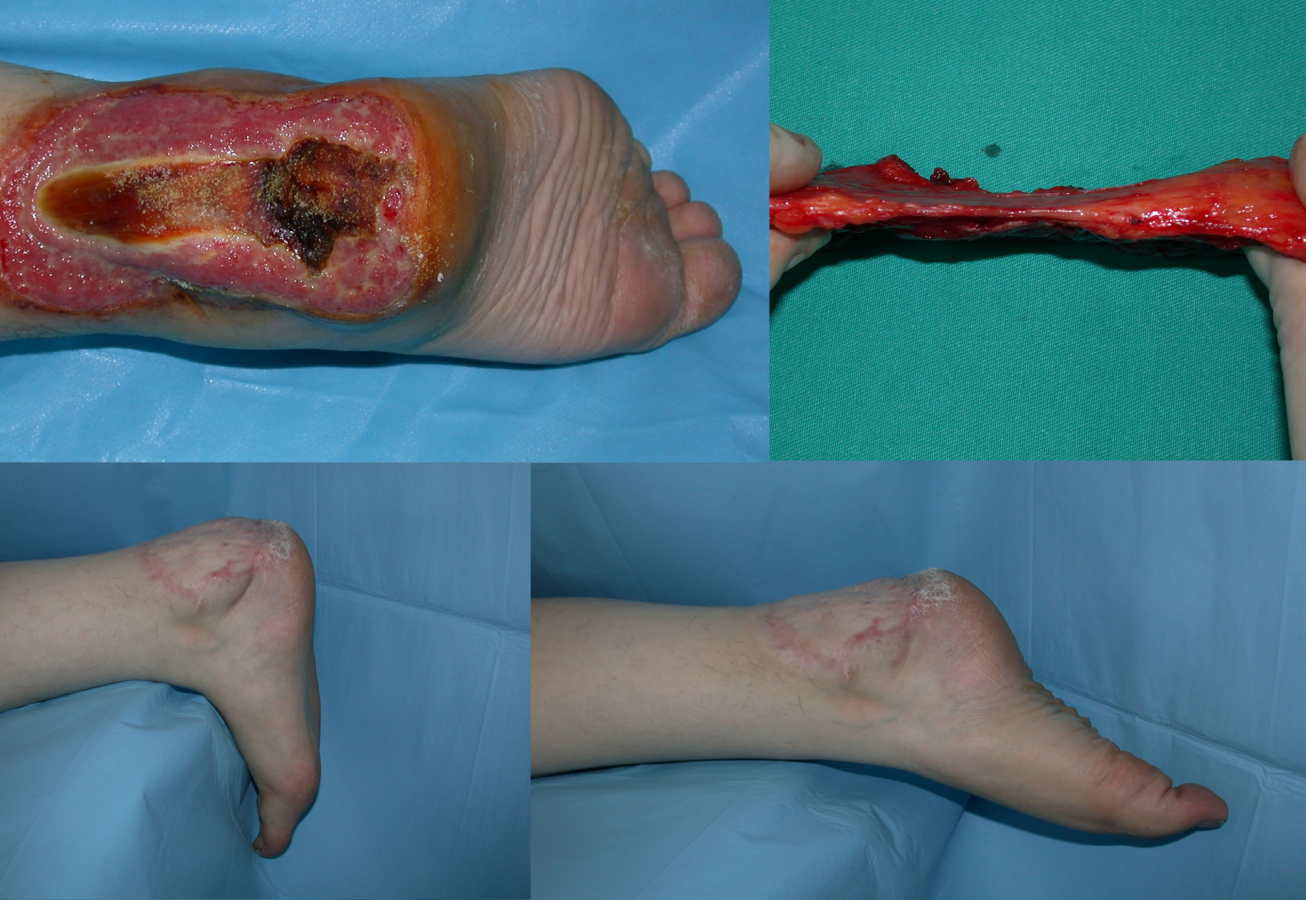
Case 9: A 25-year-old male patient who has a tissue defect exposing the right Achilles tendon and calcaneus due to a burn caused by an electrical heater used during the operation.

**Figure 2:**
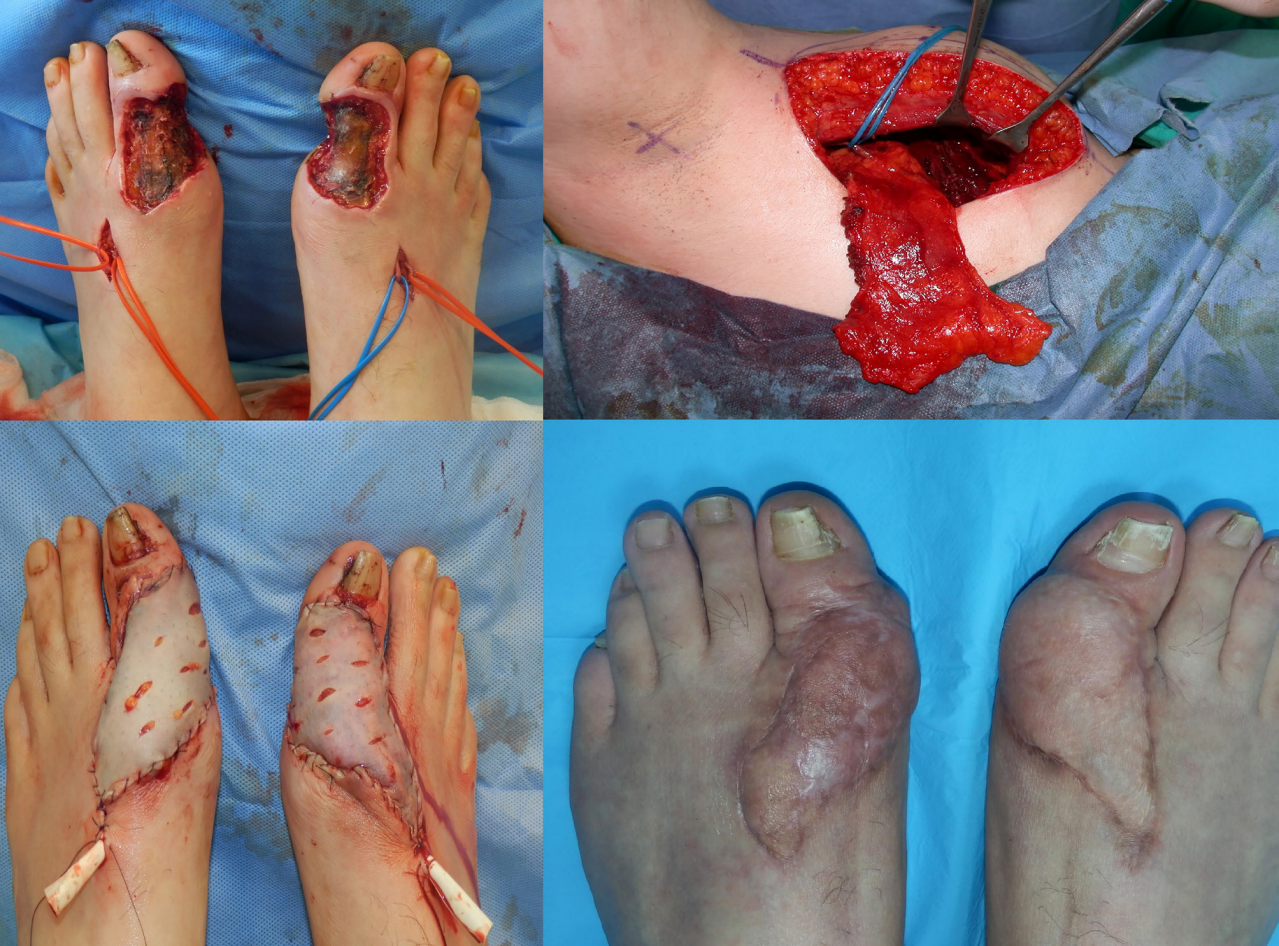
Case 4: A 27-year-old male patient who has full-thickness skin necrosis to the bone on both toes due to the local anesthetic injections administered before an operation for bilateral ingrown toenails.

**Figure 3:**
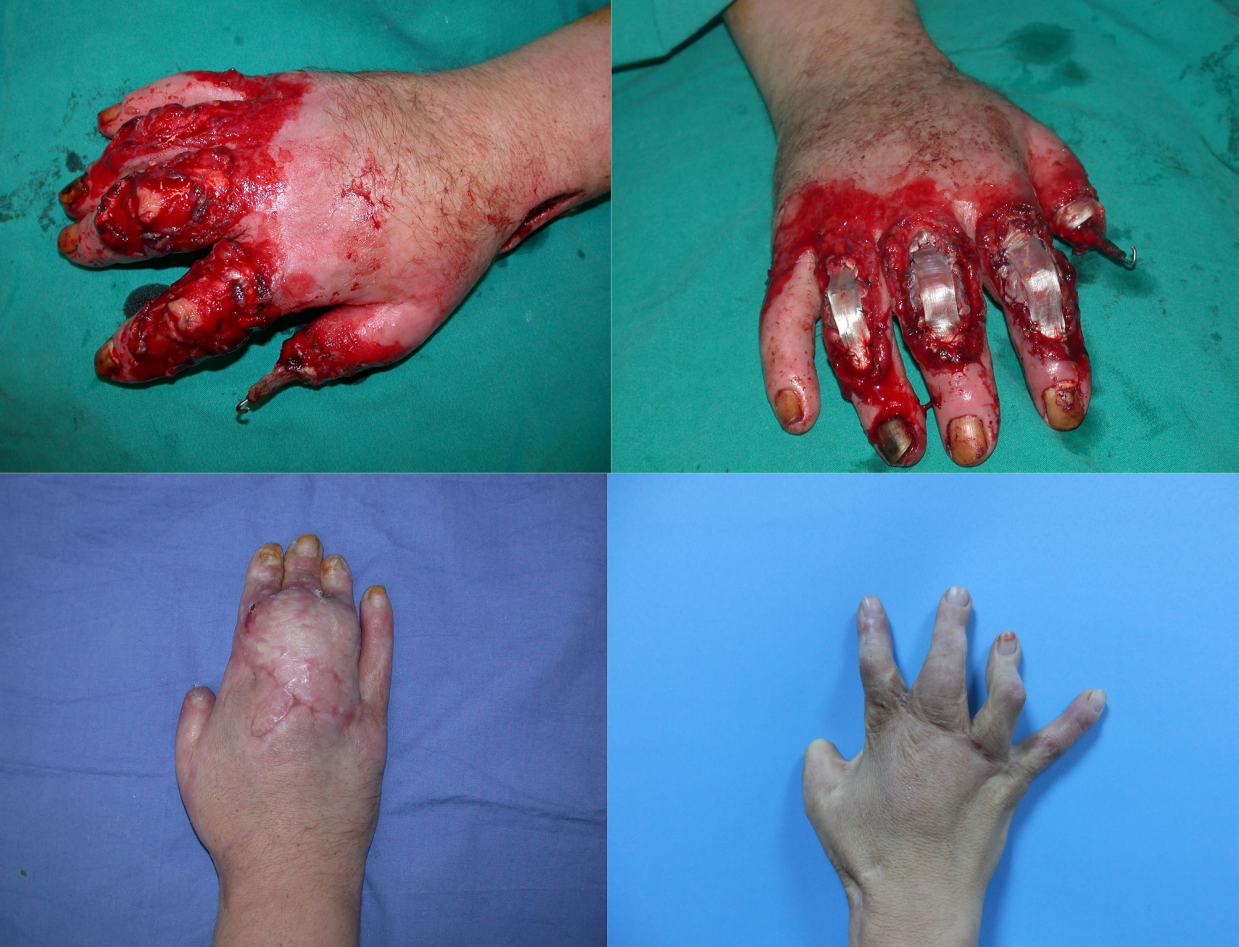
Case 7: A 30-year-old female patient who has full-thickness tissue defects to the bone that expose the distal phalanx of the thumb and dorsal surfaces of the second, third, and fourth fingers of the right hand due to a traffic accident. Reconstruction of the extensor mechanisms with tensor fascia lata tendon grafts.

Some cases are shown in Figures 1, 2, and 3.

## 4. Discussion

If shallow extremity defects exposing vital structures as bone, tendon, vessels, and nerves are too large to close primarily, they are not appropriate for reconstruction with skin grafts, and thus flaps remain the sole option for repair. The required features of the flap for ideal functional and aesthetic outcomes include criteria such as allowing gliding movement, being a pliable and thin structure, not preventing postoperative functions and movements, and not creating a level between the flap and surrounding tissues, as well as good color and texture match. It is also expected that the flap should not cause serious donor site morbidity and it should not leave a hidden scar. Unfortunately, no pedicled flap option that meet these purposes is readily available, except for the free flaps.

The radial forearm skin [1] and fascial flaps [5] have been successfully used in reconstruction of shallow extremity defects. However, scarification of a major vessel and the leaving of a conspicuous scar in the donor area are the main disadvantages of the method. Temporoparietal fascial flaps [4] cannot be harvested in large dimensions and leave poor donor site scars resulting in alopecia. Lateral arm fascial flaps [6] are not popular due to their thick structure, limited dimensions, and damage to the donor area.

Wintsch and Helaly first defined the thoracodorsal vessel-based free fascial flap on the serratus anterior muscle in 1986 and used it successfully in 4 wrist defects [8]. Schwabegger et al. applied the same flap in 3 cases for hands and firstly introduced the term “free serratus fascial flap” (FSFF) [9]. Fassio et al. used the FSFF together with the latissimus dorsi flap and scapular bone flap in reconstruction of hand dorsum defects in a chimeric style [10]. Fluegel et al. obtained satisfactory results in the postoperative hand functions and aesthetic appearances in a series of 11 cases of FSFF [11]. The serratus fascia was also used successfully in finger degloving injuries [12].

The FSFF also has become an alternative for reconstruction of lower extremity shallow defects, as well. Karşıdağ et al. applied serratus flap as fascia, muscle, and chimeric flaps associated with the latissimus dorsi muscle [13]. Meland and Weimar used the same flap, with the name of “lateral thoracic fascial flap”, in 4 distal extremity defects and reported 1 case of necrosis [14]. Ferreira et al. closed the exposed Achilles tendon with the FSFF, similar to our 2 cases [15]. Our study of 12 FSMFFs that covered both extremities is one of the largest series of the literature.

Ugurlu et al. used this flap as a bared flap in a case of a pharyngo-esophageal defect and reported that the FSFF was successfully covered with mucosa after the operation [16]. Ugurlu et al. also reconstructed large palatomaxillary defects with the FSFF combined with scapular bone in their series of 9 cases [17].

The FSFF has certain advantages, such as being easier to reach, its constant vascular pedicle without any variation, no need to sacrifice a major vessel, not leaving a hidden and short scar, and harvesting in large dimensions, as well as allowing simultaneous use with the surrounding tissues like the latissimus dorsi muscle and scapular bone. It is also possible to elevate bilateral FSFFs simultaneously in multiple defects. There is often no need for position change within the operation to take the flap and it is possible for two teams to work at the same time.

Although the FSFF has a constant vascular anatomy and a low rate of vascular variation [18], flap losses of 16.6% [9], 25% [10], 18% [11], 11% [12], and 25% [14] have been reported in the literature. Damage of the vascular plexus during dissection may account for this. A careful, meticulous, and bleeding-controlled dissection is very important in order not to disrupt the fascial vascular plexus and to increase the safety of the blood supply. In addition, including some of the underlying serratus anterior muscle in the flap in a split fashion may increase the safety of the flap. In this study, we took the flap with a very thin muscle tissue in order not to damage the subfascial plexus. Thus, we aimed to increase the flap’s success without causing important cosmetic and functional problems. The success rate of FSMFF in our study was 100%.

In conclusion, in reconstruction of shallow extremity defects exposing vital structures such as tendon, nerve, vessel, and bone where a gliding effect is desired, the FSMFF may be an ideal option with its advantage of not sacrificing a major vessel, as well as not leaving a hidden and short donor site scar and the opportunity of harvesting in wide dimensions. Moreover, the FSMFF can also be used for the purpose of filling in moderate atrophic areas on the face or other parts of the body.
